# Detection of MCM5 as a novel non-invasive aid for the diagnosis of endometrial and ovarian tumours

**DOI:** 10.1186/s12885-020-07468-y

**Published:** 2020-10-15

**Authors:** J. Stockley, R. Akhand, A. Kennedy, C. Nyberg, E. J. Crosbie, R. J. Edmondson

**Affiliations:** 1 Arquer Diagnostics Ltd, North East Business and Innovation Centre, Wearfield, Sunderland, SR5 2TA UK; 2 Department of Obstetrics and Gynaecology, Manchester Academic Health Science Centre, Saint Mary’s Hospital, Central Manchester NHS Foundation Trust, Manchester Academic Health Science Centre, Level 5, Research Floor, Oxford Road, Manchester, M13 9WL UK; 3 Division of Cancer Sciences, Faculty of Biology, Medicine and Health, University of Manchester, Saint Mary’s Hospital, Manchester, UK

**Keywords:** Endometrial cancer, Ovarian cancer, Early detection, MCM5, Biomarker

## Abstract

**Background:**

MCM5 is a protein involved in DNA replication, facilitating cell proliferation. In normal epithelium MCM5 expression is restricted to the cells in the basal proliferative compartments, however in the presence of a tumour MCM5 positive cells are present at the surface epithelium and are shed into bodily fluids. The aim of this study was to determine the sensitivity of MCM5 as a biomarker for the detection of endometrial and ovarian cancer.

**Methods:**

Patients with known ovarian or endometrial cancers, or known benign gynaecological conditions, were enrolled. Informed consent was obtained prior to the collection of full void urine, and either a vaginal tampon (worn for 6–8 h), or a vaginal swab. Vaginal secretions were extracted from the tampon or swab, centrifuged and lysed. Urine samples were centrifuged and lysed. MCM5 levels were determined by MCM5-ELISA (Arquer Diagnostics Ltd).

**Results:**

125 patients completed the study protocol, 41 patients had endometrial cancer, 26 ovarian cancer, and 58 benign controls. All patients provided a urine sample and either a tampon or vaginal swab sample. Urine MCM5 levels were higher in cancer patients than controls (*p* < 0.0001), there was no significant difference in levels between tampon samples or vaginal swab samples in cancer patients when compared to controls.

Performance of MCM5 to discriminate cancer from benign disease was high with an area under the ROC curve of 0.83 for endometrial cancer and 0.68 for ovarian cancer. Using a cut off of 12 pg/mL, overall sensitivity for endometrial cancer was 87.8, and 61.5% for ovarian cancer with a specificity of 75.9%.

**Conclusions:**

MCM5 is a novel sensitive and specific biomarker for the detection of ovarian and endometrial tumours in urine samples, which is likely to have clinical utility as a diagnostic aid.

## Background

Ovarian and endometrial cancers, the cancers of the upper female genital tract, share many similarities [1, 2]. Combined, they represent a major disease burden with over 16,000 cases diagnosed each year in the UK. Both actually represent a collection of different histological subtypes, some of these subtypes being common to both ovarian and endometrial cancer, and both organs are in direct continuity, via the fallopian tubes and the cervix, with the lower genital tract.

To date, screening and early diagnosis of upper genital tract cancers has been challenging. Although many patients with endometrial cancer present with post menopausal bleeding this is by no means universal whilst ovarian cancer is notorious for late presentation, in part because of a lack of red flag symptoms. Screening for ovarian cancer, in the form of CA125 and transvaginal ultrasound has not been demonstrated to improve clinical outcome [3]. Furthermore diagnostic tests for patients suspected of having endometrial and ovarian disease lack specificity, leading to unnecessary surgery, and are invasive which patients find unacceptable.

There is therefore a significant unmet need to generate better tests that can be used, alone or in combination, in either a screening or a diagnostic setting.

Spontaneous shedding of cancer cells from upper genital tract gynaecological tumours, which migrate through the cervix and are secreted vaginally, may prove a critical phenomenon to aid the development of new tests.

In endometrial cancer the presence of tumour cells in the lower genital tract has been shown through a number of methods. Incidental findings of Pap smears (for cervical cancer screening) found that 20% of endometrial cancer patients have endometrial cancer cells present in the cytology of the Pap smear [4]. There have also been a number of studies which have demonstrated the detection of molecular markers in cervico-vaginal secretions of endometrial cancer patients, further strengthening the hypothesis that cancer cells are shed and secreted vaginally [5, 6].

Testing for the presence of cells is labour intensive and includes a degree of subjectivity. Conversely, molecular markers are also relatively expensive and require specialist equipment. The ideal test is one that can be replicated easily and reliably in any suitable pathology laboratory.

MCM5(mini chromosome maintenance 5) is a DNA licensing factor involved in cell proliferation, and has been previously established as an excellent biomarker in a number of malignancies, including cervical, bladder and prostate cancer [7–9]. MCM5 plays a critical role in DNA replication (reviewed in [10]) and is expressed in any cell capable of proliferation. Importantly, however, expression is lost in terminally differentiated cells. In a normal epithelial structure MCM5 positive, proliferating cells are thus restricted to the basal ‘stem-cell’ compartment, with cells at the epithelial surface being terminally differentiated (and therefore MCM5 negative). Therefore any cells lost into bodily fluids, such as urine, seminal fluid or vaginal discharge should not express MCM5. However, in the case of a malignancy whereby cell growth is uncontrolled and there is an increase in the immature-non-differentiated cells these cells can be found at epithelial surfaces and therefore may be found in bodily fluids. MCM5 can be measured using the widely available technique of ELISA.

Our purpose was to determine whether MCM5 could be detected in the urine and/or vaginal secretions from patients with gynaecological cancers and if this could be used to differentiate from patients with benign conditions.

## Methods

### Study population

Subjects and controls were enrolled into the study at Saint Mary’s Hospital, Manchester, between March 2017–July 2019, ethical approval was obtained from South Central- Oxford B Research Ethics Committee (16/SC/0643) and informed consent obtained from all patients prior to the collection of urine, tampon or swab samples. For the cancer cohort all eligible patients with a known or strong suspicion of ovarian or endometrial cancer were enrolled, whilst for the benign cohort all eligible patients with a known benign condition (namely Endometriosis, Fibroids, Polycystic Ovary syndrome (PCOS) and Post-menopausal bleeding (PMB)) were enrolled. Patients were excluded if they were virgo intacta, if they had a previous diagnosis of bladder or renal cancer, if the patient had undergone any urological instrumentation in the preceding two weeks or if the patient was currently receiving chemotherapy or radiotherapy. All patients were confirmed to have negative cervical cytology within the previous three years and all patients underwent cross sectional imaging to exclude other malignancies including urothelial malignancies. Patients were asked to provide two samples, a full void urine sample and either a vaginal swab collected by the research nurse, or a vaginal tampon worn 6–8 h prior to their appointment.

### Sample processing-urine

A minimum of 25 mL urine was collected from each patient, urine was agitated to ensure a homogenous mix and up to 50 mL was transferred into a clean centrifuge tube. Samples were centrifuged at room temperature at 1500 g for 5 min. Supernatant was discarded taking care not to disturb the cell sediment pellet and tubes were placed upside down to drain on absorbent paper. Cell sediment pellets were resuspended in an appropriate volume of ADXLYSIS buffer (Arquer Diagnostics Ltd) (10uL per mL of urine) and incubated at room temperature for 1 h before being stored at less than -20 °C.

### Sample processing-vaginal tampon

Patients were asked to wear a commercially available cardboard applicator vaginal tampon and instructed to place the tampon in their vagina for 6–8 h prior to their attendance at the clinic. The tampon was removed and placed in a 50 mL centrifuge tube and phosphate buffered saline (PBS) added. The tampon was transferred to a large syringe and compressed to release the PBS/cells from the tampon. The PBS/cells from the tampon were collected in a fresh 50 ml centrifuge tube. Tubes containing PBS/cells extracted from the syringe were centrifuged at room temperature at 1500 g for 5 min. Supernatant was discarded, taking care not to disturb the cell sediment pellet. Tubes were placed upside down on absorbent paper to drain and the cell sediment pellets were resuspended in 300 μL Lysis buffer and incubated at room temperature for 1 h before being stored at less than -20 °C.

### Sample processing-vaginal swab

Vaginal swabs were collected by the research nurse, briefly; a soft endocervical collection brush was inserted 3–5 cm into the vagina and rotated four times (two towards the left and two towards the right). The swab was then placed into 5 mL of PBS. The vaginal swab was removed from the tube and the PBS was centrifuged at room temperature at 1500 g for 5 min. Supernatant was discarded, taking care not to disturb the cell sediment pellet. Tubes were placed upside down to drain on absorbent paper and the cell sediment pellets were resuspended in 300 μL Lysis buffer and incubated at room temperature for 1 h before being stored at less than -20 °C.

### MCM5 ELISA

Patient lysates were tested with an MCM5 ELISA as per manufacturer’s instructions (Arquer Diagnostics). Briefly; 100 μL of lysate was added to each of 2 wells of the MCM5 ELISA micro-titre plate (samples and controls were run in duplicates) and incubated for 60 min at room temperature on a plate shaker (700RPM). Following incubation wells were washed 6 times with 350 μL of 1x wash buffer using an automated plate washer. 100 μL of MCM5-HRP conjugated antibody (Arquer Diagnostics Ltd., Sunderland UK) was added to each well and incubated at room temperature for 30 min prior to being washed 6 times with 350 μL of 1x wash buffer as above. 100 μL of TMB was added to each well and incubated for 30 min in the dark prior to the addition of a stop solution (0.5 M H_2_SO_4_). Optical density (OD) was measured at 450 nm and 630 nm (reference wavelength). Concentrations of MCM5 were calculated using a serial dilution standard curve of a known recombinant MCM5 control (1.3 mg/ml) with a negative control (Lysis Buffer).

### Statistical analysis

The study was carried out and reported in line with the 20 recommendations set out in the STARD 2015 guidance for diagnostic test reporting [11].

Data were analysed using MS Excel and GraphPad Prism (v7.04). The chi square test was used to assess categorical variables and receiver operating characteristic curves were generated to assess performance of the assay. The cut-off point of 12 pg/mL was determined by the manufacturer’s instructions as determined from previous studies [7]. ROC curves were generated using MCM5 level as a continuous variable and presence or absence of malignancy as a discrete variable.

Using the methodology described by Pepe et al. [12] it is possible to model the potential clinical utility of a biomarker incorporating both the incidence and possible cost benefit ratio of a test as follows:

$$ \frac{TPR}{FPR}>\frac{1-p}{p}\times r $$

In this equation, TPR is the true positive rate, FPR is the false positive rate (1-specificity) and TPR/FPR defines the *actual performance* of the test. *p* represents the prevalence of the cancer and *r* is the cost benefit ratio and the right side of the equation defines the *performance required*. Where the actual performance is greater than the performance required (i.e. where left side of equation is greater than the right) then the test is likely to have clinical utility.

## Results

### Clinical characteristics

Sixty-seven cancer patients were recruited and completed the study protocol, 41/67 with proven endometrial cancer and 26/67 with confirmed ovarian cancer. Cancer patients had a median age of 70 (IQR: 55–77) (endometrium) and 63 (IQR 52–69) (ovary) which is typical for these diseases, Table 1. There was good representation of all subtypes, grade and stages of cancer within the cohort with 29/41 (71%) endometrium and 8/26 (31%) ovarian being stage 1, Table 1.

**Table 1:** Patient Demographics of cancer cohort at time of recruitment

Characteristic	N (%) or median (interquartile range)	
	Endometrial cancer cohort	Ovarian cancer cohort
**Age (year)**	70 (57-77)	63 (52-69)
Histological subtype		
Endometrioid	24 (59)	3 (12)
Serous	6 (15)	15 (58)
Clear cell	5 (12)	1 (4)
Squamous cell	-	1 (4)
Carcinosarcoma	4 (10)	-
Adenocarcinoma NOS	-	1 (4)
Not recorded	2 (5)	2 (8)
**Grade**		
Borderline	-	3 (12)
G1	14 (34)	4 (15)
G2	10 (24)	3 (12)
G3	17 (41)	16 (62)
**FIGO stage**		
I	29 (71)	8 (31)
II	4 (10)	4 (15)
III	7 (17)	10 (38)
IV	1 (2)	1 (4)
Not recorded	-	3 (12)

Fifty-eight control patients were also recruited and completed the study protocol. These were patients attending gynaecology clinics for benign disease. Median age was younger than the cancer patients (49) (IQR 39–56) and patients had a variety of benign conditions, Table 2, but none developed cancer within 6 months following recruitment.

**Table 2:** Patient Demographics of benign cohort at time of recruitment

Characteristic	N(%) or median (interquartile range)
	Benign cohort
**Age (years)**	49 (39-56)
**Gynaecological Condition**	
Endometriosis	10 (17)
Fibroids	13 (22)
Post menopausal bleeding	18 (31)
Ovarian Cyst	4 (7)
Polycystic ovaries	7 (12)
Infarcted fibroma	1 (2)
Mature Cystic Teratoma	2 (3)
Not recorded	3 (5)

A further 22 patients were approached but either declined to participate or failed to complete the study protocol. Specifically 3 patients stated they were unable, or not prepared, to wear a tampon for the required eight hours.

### MCM5 levels in the urine sediment of cancer patients compared to controls

**Fig. 1:**
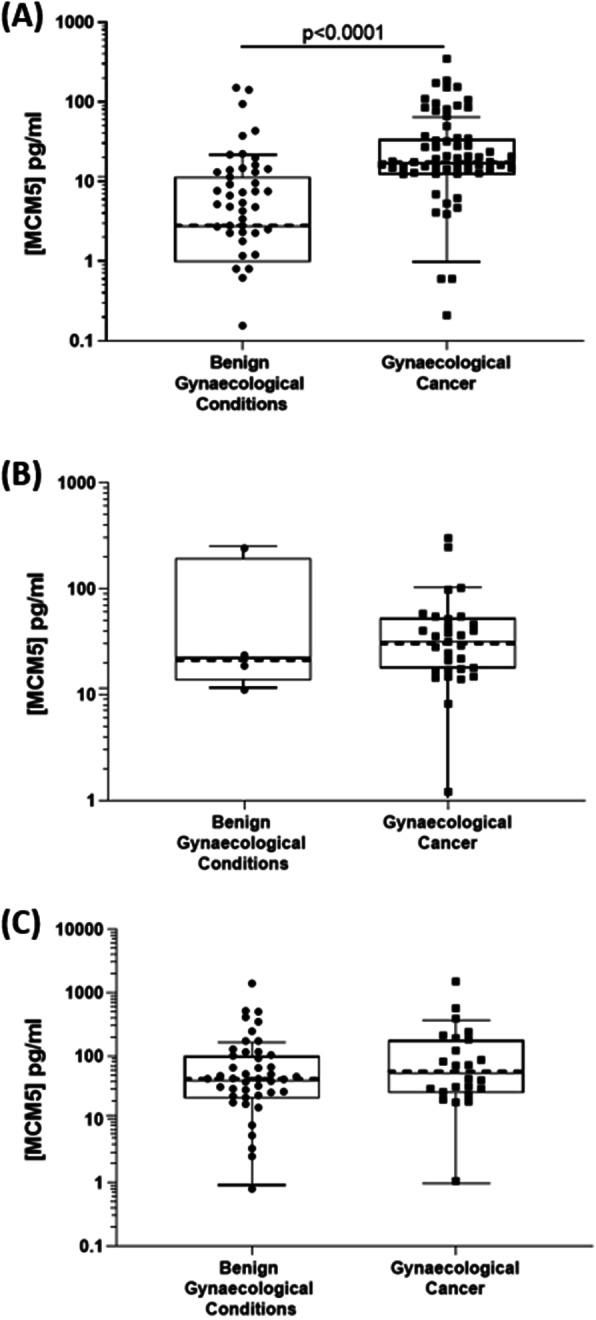
MCM5 level in Benign vs Cancer patients detected in (**a**) Urine, (**b**) Vaginal Tampon, (**c**) Vaginal Swab

MCM5 levels were tested in samples (urine sediment, plus vaginal swab or tampon) from all 67 cancer patients and all 58 control patients. Median levels were higher in the urine samples from cancer patients (17.60 pg/mL) compared to controls (2.81 pg/mL), *p* < 0.0001, Fig. 1a. Although median levels of MCM5 were slightly higher in the swab and tampon samples from cancer patients compared to controls these were non significant, Fig. 1b and c.

### Urine sediment MCM5 as a discriminator of both endometrial and ovarian cancer

**Fig. 2:**
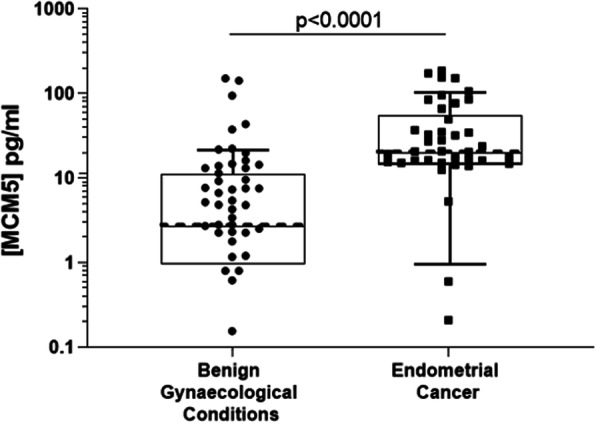
MCM5 levels in urine sediment in Benign vs Endometrial Cancer

Comparing MCM5 levels in urine sediment samples from patients with endometrial cancer with controls showed a highly significant increase in cancer cases (median 20.57 pg/mL for cancer and 2.81 pg/mL for controls), Fig. 2. Using a pre defined cut off of 12 pg/mL resulted in a correct classifier in 80/99 (80.1%) cases, Table 3. Overall sensitivity for endometrial cancer was 87.8% and remained high for both low grade disease (85.7, 95% CI: 57.2–98.2%) and low stage disease (86.2, 95% CI: 68.3–96.1%), Table 4.

**Table 3:** Contingency table for test performance for endometrial cancer

	Endometrial cancer	
MCM5 result	Positive	Negative
Positive	36	14
Negative	5	44

**Table 4:** Diagnostic performance of MCM5 urine test as a biomarker by tumour type

	Ovarian cancer	
MCM5 result	Positive	Negative
Positive	16	14
Negative	10	44

**Fig. 3:**
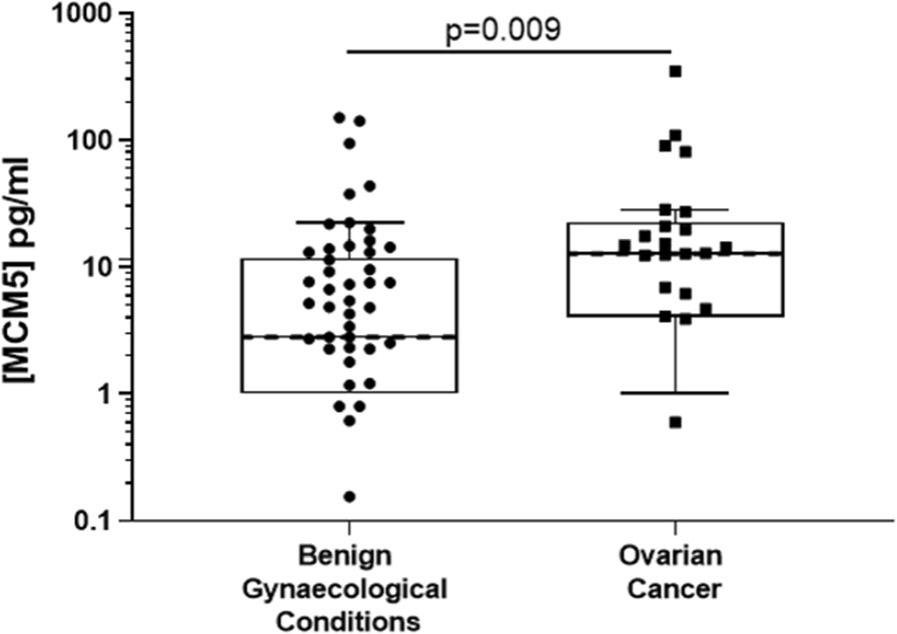
MCM5 levels in urine sediment in Benign vs Ovarian Cancer

Comparing MCM5 levels in urine sediment samples from patients with ovarian cancer with controls showed a highly significant increase in cancer cases (median 12.85 pg/mL for cancer and 2.81 pg/mL for controls), Fig. 3. Using a cut off of 12 pg/mL resulted in a correct classifier in 60/84 (71.4%) cases, Table 5. Overall sensitivity for ovarian cancer was 61.5% but interestingly was higher for low grade disease (71.4, 95%CI: 29.0–96.3%) and low stage disease (75, 95%CI: 34.9–96.8%). Two-thirds of the borderline ovarian samples had levels greater than 12 pg/mL classifying them as positive.

**Table 5:** Contingency table for test performance for ovarian cancer

Type of tumour(n)	Sensitivity(%)	Specificity (%)
	(95%CI(%))	(95%CI(%))
**Endometrial cancer (41)**	87.8 (73.8-95.9)	75.9 (62.8-86.1)
Low grade(14)	85.7 (57.2-98.2)	-
High Grade(27)	88.9 (70.8-97.7)	-
Stage I (29)	86.2 (68.3-96.1)	-
Stage II and above(12)	91.7 (61.5-99.8)	-
**Ovarian cancer(26)**	61.5 (40.6-79.8)	75.9 (62.8-86.1)
Low Grade(7)	71.4 (29.0-96.3)	-
High Grade(19)	57.9 (33.5-79.8)	-
Stage I(8)	75.0 (34.9-96.8)	-
Stage II and above(15)	53.3 (26.6-78.7)	-

Specificity for the MCM5 test in this cohort was 75.9% (95%CI: 62.8–86.1%). None of the benign conditions tested showed an increase in MCM5 levels compared to the remaining controls (data not shown).

### Potential clinical utility for the MCM5 test

**Fig. 4:**
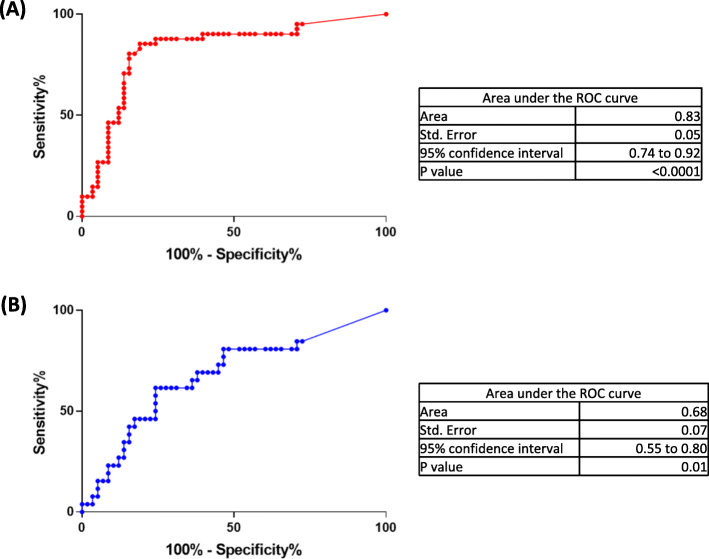
Receiver Operator Curve showing performance of MCM5 as a diagnostic test for (**a**) Endometrial Cancer and (**b**) Ovarian Cancer

Overall performance of the MCM5 test was higher for endometrial cancer with an area under the ROC curve of 0.83 compared to 0.68 for ovarian cancer, Fig. 4, suggesting a good performance for MCM5 as a biomarker, at least for endometrial cancer. However clinical utility of a biomarker is dependent upon not only performance of the biomarker but also the incidence/prevalence of the condition being tested, and the cost benefit ratio of employing the assay.

Using the Pepe model (see methods) MCM5 *actual performance* was therefore compared to the *performance required* for two clinical settings, namely the asymptomatic screening population and a diagnostic testing population (following referral for symptoms).

For the asymptomatic screening population age standardised incidence of disease was used for prevalence (33/100,000 for endometrial and 40/100,000 for ovarian [13]), whilst for the diagnostic testing population *p* = 3% was used, given that this is the minimum threshold required for referral by NICE [14]. r was set at 0.1, the value given for a scenario of 10 patients being subjected to unnecessary tests for each cancer case diagnosed [12], a value widely accepted in the literature [15].

As expected, *performance required* for a screening test was higher (302.9 for endometrial and 249.9 for ovarian) than for a diagnostic test (3.2 for endometrial and 3.2 for ovarian). *Actual performance* of the MCM5 test (3.6 for endometrial and 2.5 for ovarian) was therefore substantially lower than required for a screening test but broadly equivalent to that needed for a diagnostic test, Table 6.

**Table 6:** Comparison of performance required for a screening or diagnostic test against actual performance of MCM5 test

	Actual performance^a^	Performance required^1^	
		Screening	Diagnostic
Endometrial	3.64	302.9	3.2
Ovarian	2.55	249.9	3.2

^a^calculated from [13], see text for details a test is likely to have clinical utility if: actual performance > performance required

## Discussion

Here we have shown that the DNA licensing factor MCM5 can be detected in the urine sediment of patients with gynaecological cancers at levels in excess of those found in patients with benign gynaecological conditions. Urine sediment testing was more successful than vaginal tampon or vaginal swab testing and was more acceptable to patients. Of particular interest was that the detection of MCM5 was maintained, and possibly improved, in patients with low stage and low grade disease, which is encouraging for clinical use where detection of early stage, low grade disease is critical.

Biomarkers have clinical utility in both the asymptomatic population as screening tests and as part of the diagnostic pathway for patients with symptoms. Performance accuracy requirements are different for these two settings with asymptomatic screening in particular requiring tests with extremely high specificities to avoid over investigation [15]. Whilst the MCM5 test outlined here does not, at this stage, appear to be associated with the specificity required for a screening test it may have an important role, alone, or in conjunction with other tests, as part of the diagnostic process. Particularly, as it is non-invasive, it may have an important role early in the pathway as a “rule out” test to avoid unnecessary further invasive testing in those who are at low risk of harbouring disease.

To this end, whilst this is an early phase diagnostic accuracy study it benefits from including benign gynaecological conditions in the control arm as these patients are often represented in a diagnostic clinic and require differentiation from those who have cancer. However the numbers in the current study remain small and further studies including larger numbers and replicating more closely the clinical setting are justified. Specifically, future studies should replicate the prevalence of cancer more accurately than in the current dataset.

The MCM5 test has previously been shown to have utility in bladder and prostate cancer [7, 8] and has been shown to have signal in cervical intraepithelial neoplasia and Barrets oesophagus. However this is the first time that it has been shown to be of use in gynaecological cancers. The MCM5 test is an easy-to-perform ELISA test, compatible with general laboratory equipment available in most hospital laboratories, and can provide results within 3 h, without the need for a pathologist, making it a potential low-cost alternative or adjunct to cytology, which remains non standardised for gynaecological cancers, or other methodologies. It is likely that the use of MCM5 as an adjunctive test will improve sensitivity and, when combined with other tests in this way, be associated with appropriate specificity.

It is perhaps not surprising that the MCM5 test performed better as a test for endometrial cancer than for ovarian cancer. Lesions in the endometrial cavity are located closer to the vagina and cells shed from these tumours are more likely to be detected in vaginal fluid or urine compared to tumours located higher in the genital tract. However, it is now widely accepted that many high grade serous cancers arise in the fallopian tube rather than the ovary and thus the MCM5 test may have use in at least some of these cancers. Utilising the MCM5 test for this indication should not be ignored in future studies.

The wearing of a tampon was unpopular with patients and furthermore was associated with high readings in the control patients with benign disease, which resulted in the poor performance of MCM5 as a discriminator in this settingThis may be related to the tampon causing trauma to the vaginal mucosa, thus exposing basal cells that normally express MCM5. Further studies should therefore be limited to assessment of urine sediment but it will be important to ensure that samples collected in such studies do not follow instrumentation, or indeed examination, of the lower genital tract.

## Conclusions

Here we have demonstrated for the first time, that a simple, non-invasive test based upon the MCM5 biomarker may have clinical utility in the early diagnosis of female upper genital tract cancers.

## Data Availability

Not applicable.

## Abbreviations

CI: Confidence interval
DNA: Deoxyribonucleic acid
ELISA: Enzyme linked immunosorbent assay
FPR: False positive rate
HRP: Horse radish peroxidase
IQR: Inter quartile range
MCM5: Mini chromosome maintenance 5
NICE: National Institute for Health and Clinical Excellence
OD: Optical density
PBS: Phosphate buffered saline
PCOS: Polycystic ovarian syndrome
PMB: Post menopausal bleeding
ROC: Receiver operating characteristic
TMB: Tris magnesium sulphate buffer
TPR: True positive rate
UK: United Kingdom
